# Unveiling the signals from extremely noisy microseismic data for high-resolution hydraulic fracturing monitoring

**DOI:** 10.1038/s41598-017-09711-2

**Published:** 2017-09-20

**Authors:** Weilin Huang, Runqiu Wang, Huijian Li, Yangkang Chen

**Affiliations:** 1 State Key Laboratory of Petroleum Resources and Prospecting, China University of Petroleum-Beijing, Beijing, 102249 China; 2 Modeling and Imaging Laboratory, Earth and Planetary Sciences, University of California at Santa Cruz, Santa Cruz, 95064 California USA; 3 SINOPEC Exploration and Production Research Institute, Beijing, 100083 China; 4 Jackson School of Geosciences, The University of Texas at Austin, University Station, Box X, Austin, TX 78713-8924 USA; 5 Present Address: National Center for Computational Sciences, Oak Ridge National Laboratory, Oak Ridge, TN 37831-6008 USA

## Abstract

Microseismic method is an essential technique for monitoring the dynamic status of hydraulic fracturing during the development of unconventional reservoirs. However, one of the challenges in microseismic monitoring is that those seismic signals generated from micro seismicity have extremely low amplitude. We develop a methodology to unveil the signals that are smeared in the strong ambient noise and thus facilitate a more accurate arrival-time picking that will ultimately improve the localization accuracy. In the proposed technique, we decompose the recorded data into several morphological multi-scale components. In order to unveil weak signal, we propose an orthogonalization operator which acts as a time-varying weighting in the morphological reconstruction. The orthogonalization operator is obtained using an inversion process. This orthogonalized morphological reconstruction can be interpreted as a projection of the higher-dimensional vector. We first test the proposed technique using a synthetic dataset. Then the proposed technique is applied to a field dataset recorded in a project in China, in which the signals induced from hydraulic fracturing are recorded by twelve three-component (3-C) geophones in a monitoring well. The result demonstrates that the orthogonalized morphological reconstruction can make the extremely weak microseismic signals detectable.

## Introduction

It has been shown that microseismic monitoring has a significant potential to characterize physical processes related to fluid injections and extractions in hydrocarbon and geothermal reservoirs^1,2^. In general the microseismicity is recorded by downhole or shallow surface geophone arrays, which offers the significant advantages of being sufficiently close to the fracture and being unaffected by the free surface^3^. There are two main physical processes involved in hydraulic fracturing: 1) penetration of the injected fluid into the pre-existing cracks and pore spaces when the injection pressure is lower than the minimum compressive stress, and 2) opening of new fractures when the injection pressure is high enough. The events generated during injection and also after injection can occur over hours^4^. Localization of the associated microseismic events enables imaging of the fracture network. This technique has been widely studied and applied in petroleum and gas exploration^1,5–11^, and mining engineering^12–15^. However, an inevitable problem existing in the microseismic monitoring is that the energy stimulated from the hydraulic fracturing is extremely weak, compared with the background noise^16^. The weak signal is easily masked, resulting in loss of microseismic events. A poor signal-to-noise ratio (S/N) can lead to unauthentic arrival time-picks^17^ and localization of microseismic events^18^. All of these will negatively affect the performance of microseismic monitoring and resulted fracture imaging^19^, as well as solving source mechanisms^20^. Improving the S/N will ultimately improve the microseismic event detection. In microseismic monitoring, the most commonly used method for attenuating background noise and detecting weak signal is frequency filtering^21^. However, frequency filtering typically fails in separating noise and signal when they share the same frequency band. Researchers put a lot of effort into the noise suppression problem^22^, and developed different techniques using different approaches such as: median filtering^23^, various kinds of mathematical transform based approaches^24–26^, and matrix completion based approaches^27,28^. In addition, Kong *et al*. develop ed a nonlinear signal detector, which passes only signals showing spatial coherence and having slowness within an allowed range. Schimmel and Paulssen^30^ use d an instantaneous phase based amplitude-unbiased coherency measure, weighting the samples of an ordinary, linear stack, to detect weak signals in global seismology. Gibbons and Ringdal^31^ illustrated the power of an array-based waveform correlation approach by detecting the low magnitude seismic events in the 1997 August 16 Kara Sea event. Mousavi *et al*.^32^ proposed a simultaneous microseismic denoising and onset detection technique based on the synchrosqueezed continuous wavelet transform and custom thresholding of single-channel data. Mousavi and Langston^33^ designed fast algorithm for noise level estimation and noise reduction of micro-seismic data, using minimally controlled recursive averaging and neighborhood shrinkage estimators.

The denoising approach of this paper is based on mathematical morphological decomposition and reconstruction^34,35^. Mathematical morphology is a nonlinear methodology for the analysis and processing of geometrical structures. Matheron^34^ describe d the random set integral geometry theory and topological logic theories thoroughly and set up a consistent foundation for mathematical morphology. Later on, Serra^35^ suggested the theory and method of mathematical morphology which was widely applied in two-value image processing. Then, Koskinen *et al*.^36^ introduce d the soft mathematical operations, which can maintain most of the properties of standard morphological operations. Sinha and Dougherty^37^ developed a generalization of binary mathematical morphology based on fuzzy set theory, in which images are modeled as fuzzy subsets of the Euclidean plane or Cartesian grid, and the morphological operations are defined in terms of a fuzzy index function. The mathematical morphological filtering (MMF) was first introduced into seismic data processing by Wang *et al*.^38^. Unlike traditional methods in seismic data processing, the basis of MMF are logical operation and set theory, which can provide us a tool to process signal over the complete frequency bandwidth, improving the S/N and maintaining the resolution. Later, this method rapidly developed and was widely applied in seismic data processing. For example, Li *et al*.^21^ proposed a compound top-hat filter (CTF) extracting the large-scale information by combining opening and closing operations, and subsequently subtracting it from the microseismic data.

In this paper, we further develop a seismic application of the mathematical morphology and propose a multi-scale morphological decomposition based method to unveil weak signal in microseismic monitoring. In order to unveil weak signal, an orthogonalization operator is proposed and introduced into the process of multi-scale morphological reconstruction. The mathematical nature of the proposed orthogonalization operator is a projection operator that projects the input signal on a sub-space spanned by several selected morphological basis vectors. The assumption for this approach is that the weak signal is orthogonal to the background noise. Unlike the traditional morphological reconstruction approaches, the orthogonalized morphological reconstruction transforms the reconstruction problem into an inversion problem. However, like most of the inversion problems in geophysics, this inversion problem is ill-posed. A regularization (or a penalty) term is necessary to optimally stabilize the objective function. In this study we use the shaping regularization approach^39^ that is more convenient for solving the inversion problem in orthogonalized morphological reconstruction compared to other regularization technique such as Tikhonov’s method^40^. In the following sections, after explaining the methodology we first conducts synthetic data experiments to test the performance of the proposed orthogonalized morphological reconstruction approach. Then the proposed approach is applied to a real 3-C microseismic data set. Compared with the state-of-the-art algorithms, the proposed approach demonstrates a superior performance.

## Morphological Decomposition

Mathematical morphology^34,35^, is a well known nonlinear image processing method, which was originally motivated from the research of the relation between the penetrability of a porous medium and its lamination. It starts as a set theoretical approach for the analysis of geometrical structures but can also deal with both function and set in the Euclidean space. A morphological operation is the interaction of an objective set or function with another set or function called structuring element (SE). The morphological scale of the SE determines the scale information of the signal that is extracted under such an operation. The morphological scale can be conceptually understood as the relative structure. Let ${\bf{d}}$ be a seismic trace and ${\bf{f}}\subseteq {\mathbb{R}}$ be a set of amplitude values. The value of a sample $t$ in ${\bf{d}}$ is represented by $d(t)\in {\bf{f}}$. The morphological dilation ${\varphi }_{b}({\bf{d}})$ and erosion ${\varphi }_{b}({\bf{d}})$ are the morphological operations that process ${\bf{d}}$ with the SE $b(\tau )$ as^41^:

1 $${\varphi }_{b}({\bf{d}})=\mathop{\vee }\limits_{\tau }b(\tau )+d(t-\tau ),$$

2 $${\phi }_{b}({\bf{d}})=\mathop{\wedge }\limits_{\tau }b(\tau )-d(\tau -t),$$

where $\vee$ denotes supremum, and $\wedge$ denotes infimum. Both $t$ and $\tau$ are samples. It can be seen that the morphological dilation is an operation that “grows” or “thickens” the object, while the morphological erosion is an operation that “shrinks” or “thins” the object. The sequential combination of the morphological erosion (or dilation) and morphological dilation (or erosion) creates the morphological opening ${\chi }_{b}({\bf{d}})$ (or closing ${\psi }_{b}({\bf{d}})$) as:

3 $${\chi }_{b}({\bf{d}})={\varphi }_{b}({\phi }_{b}({\bf{d}})),$$

4 $${\psi }_{b}({\bf{d}})={\phi }_{b}({\varphi }_{b}({\bf{d}}\mathrm{)).}$$

We now use morphological opening and closing to represent data ${\bf{d}}$. Consider $\{{\chi }_{{b}_{k}}({\bf{d}})\}$, $k\in \mathrm{[1,}\,K]$ and $\{{\psi }_{{b}_{k}}({\bf{d}})\}$, $k\in \mathrm{[1,}\,K]$, two indexed families of morphological opening and closing, respectively. Typically, the index $k$ denotes the morphological scale. Whereupon, ${\bf{d}}$ can be represented as:

5 $${\bf{d}}=\sum _{k\mathrm{=1}}^{K}[{\chi }_{{b}_{k-1}}({\psi }_{{b}_{k-1}}({\bf{d}}))-{\chi }_{{b}_{k}}({\psi }_{{b}_{k}}({\bf{d}}))]+{\chi }_{{b}_{K}}({\psi }_{{b}_{K}}({\bf{d}})),$$

or

6 $${\bf{d}}=\sum _{k\mathrm{=1}}^{K}[{\psi }_{{b}_{k-1}}({\chi }_{{b}_{k-1}}({\bf{d}}))-{\psi }_{{b}_{k}}({\chi }_{{b}_{k}}({\bf{d}}))]+{\psi }_{{b}_{K}}({\chi }_{{b}_{K}}({\bf{d}}\mathrm{)).}$$

Equations (5) and (6) can also be written as:

7 $$\begin{array}{ll}{\bf{d}} & =\frac{1}{2}({\bf{d}}+{\bf{d}})\\  & =\frac{1}{2}\{{\psi }_{{b}_{K}}({\chi }_{{b}_{K}}({\bf{d}}))+{\chi }_{{b}_{K}}({\psi }_{{b}_{K}}({\bf{d}}))\\  & \quad +\sum _{k\mathrm{=1}}^{K}[{\chi }_{{b}_{k-1}}({\psi }_{{b}_{k-1}}({\bf{d}}))-{\chi }_{{b}_{k}}({\psi }_{{b}_{k}}({\bf{d}}))+{\psi }_{{b}_{k-1}}({\chi }_{{b}_{k-1}}({\bf{d}}))-{\psi }_{{b}_{k}}({\chi }_{{b}_{k}}({\bf{d}}\mathrm{))]\}.}\end{array}$$

**Figure 1:**
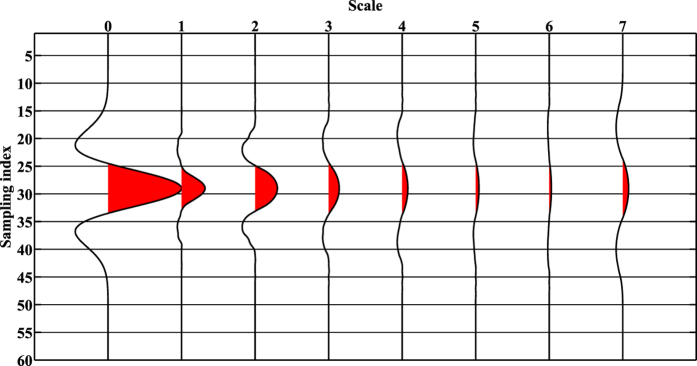
Multi-scale morphological decomposition of a Ricker wavelet.

So far, the initial data **d** is represented by an additive decomposition with *K* + 1 scales. Figure 1 gives an example of the morphological decomposition of a Ricker wavelet with 7 scales. The 1st trace (scale 0) is the initial wavelet. The 2*nd*–8th traces are the 7 scale components.

## Traditional morphological reconstruction

For convenience, let:

8 $${{\bf{c}}}_{k}=\{\begin{array}{ll}\frac{1}{2}\{{\chi }_{{b}_{k-1}}({\psi }_{{b}_{k-1}}({\bf{d}}))-{\chi }_{{b}_{k}}({\psi }_{{b}_{k}}({\bf{d}}))+{\psi }_{{b}_{k-1}}({\chi }_{{b}_{k-1}}({\bf{d}}))-{\psi }_{{b}_{k}}({\chi }_{{b}_{k}}({\bf{d}}))]\}, & \,k\in \,\mathrm{[1,}\,K],\,\\ \frac{1}{2}\{{\psi }_{{b}_{K}}({\chi }_{{b}_{K}}({\bf{d}}))+{\chi }_{{b}_{K}}({\psi }_{{b}_{K}}({\bf{d}}))\}, & \,k=K+\mathrm{1,}\,\end{array}$$

where ${{\bf{c}}}_{k}$, $k\in \mathrm{[1,}\,K+\mathrm{1]}$, are the morphological multi-scale components. The value of a sample *t* in ${{\bf{c}}}_{k}$ is represented by ${c}_{k}(t)\in {\bf{f}}$. The nature of the multi-scale morphological decomposition is to decompose the discrete data set ${\bf{d}}$ into a series of primary subsets ${{\bf{c}}}_{k}$, which satisfies that:

9 $${\bf{d}}=\underset{k\mathrm{=1}}{\overset{K+1}{\cup }}{{\bf{c}}}_{k},$$

10where ∅ denotes empty set. The reconstruction of data by ${{\bf{c}}}_{k}$ can be represented as:

11 $$\sum _{{{\bf{c}}}_{k}\in {\bf{E}}}[{{\boldsymbol{\sigma }}}_{k}{{\bf{c}}}_{k}]={\bf{d}},$$

where **E** is a subset of ${\{{{\bf{c}}}_{k}\}}_{k\in \mathrm{[1,}K]}$, that ${\bf{E}}\underline{\underline{\subset }}{\{{{\bf{c}}}_{k}\}}_{k\in \mathrm{[1,}K]}$. Constant ${{\rm{\sigma }}}_{k}\in \mathrm{[0,}\,\mathrm{1]}$ is the weighting coefficient that controls energy from different scale components. This decomposition allows for full reconstruction of the original data, when ${\bf{E}}=\{{{\bf{c}}}_{k}{\}}_{k\in \mathrm{[1,}K]}$ and ${{\rm{\sigma }}}_{k}\equiv 1$.

## Orthogonalized morphological reconstruction

In traditional morphological reconstruction, the weighting coefficient ${{\rm{\sigma }}}_{k}$ is chosen manually, which makes the reconstruction subjective. In addition, it is difficult to choose an appropriate weighting coefficient, and the choosing process costs a lot of time and manual endeavor. For weak signal detection, we define the orthogonalized morphological reconstruction, by changing ${{\rm{\sigma }}}_{k}$ from simple constant to a more flexible operator, in other words, allowing ${{\rm{\sigma }}}_{k}$ to change with *t*:

12 $${\rm{\arg }}\mathop{{\rm{\min }}}\limits_{{\sigma }_{k}(t)}\sum _{{{\bf{c}}}_{k}\in {\bf{E}}}{\Vert {{\boldsymbol{\sigma }}}_{k}(t){c}_{k}(t)-d(t)\Vert }_{F}^{2},$$

where $\parallel \cdot {\parallel }_{F}^{2}$ represents the squared Frobenius norm of a function. Equation (12) can be also represented as:

13 $${\rm{\arg }}\mathop{{\rm{\min }}}\limits_{{{\boldsymbol{\Sigma }}}_{k}}\sum _{{{\bf{c}}}_{k}\in {\bf{E}}}{\Vert {{\boldsymbol{\Sigma }}}_{k}{{\bf{c}}}_{k}-{\bf{d}}\Vert }_{F}^{2},$$

where ${{\boldsymbol{\Sigma }}}_{k}$ is a diagonal matrix composed by ${{\boldsymbol{\sigma }}}_{k}(t)$: ${{\boldsymbol{\Sigma }}}_{k}=diag({{\boldsymbol{\sigma }}}_{k}(t))$. Thus, the orthogonalized morphological reconstruction holds as:

14 $$\sum _{{{\rm{c}}}_{k}\in {\bf{E}}}[{{\boldsymbol{\Sigma }}}_{k}{{\bf{c}}}_{k}]=d\mathrm{.}$$

**Figure 2:**
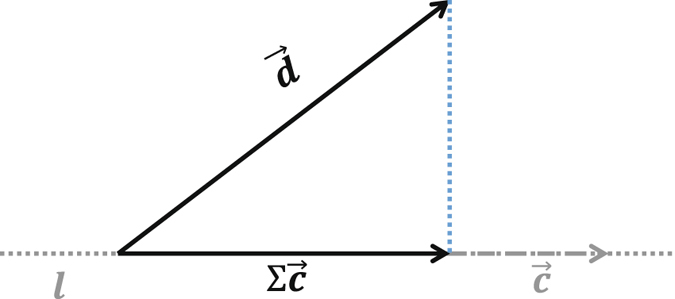
A geometrical interpretation of the orthogonalization operator.

The geometrical nature behind equation (13) is a projection of the higher-dimensional vector, i.e., the initial data ${\bf{d}}$, on a lower-dimensional space spanned by several selected morphological basis vectors. Figure 2 gives a diagrammatic drawing. Vector $\overrightarrow{{\bf{c}}}$ is on the line $l$. Operation ${\boldsymbol{\Sigma }}$ is a stretching transformation acting on vector $\overrightarrow{{\bf{c}}}$. Equation (13) is actually to find the projection of vector $\overrightarrow{{\bf{d}}}$ on line $l$ in the least-squares sense. Thus, an orthogonal decomposition of $\overrightarrow{{\bf{d}}}$ holds as:

15 $$\overrightarrow{{\bf{d}}}={\boldsymbol{\Sigma }}\overrightarrow{{\bf{c}}}+(\overrightarrow{{\bf{d}}}-{\boldsymbol{\Sigma }}\overrightarrow{{\bf{c}}}),$$

16 $$\overrightarrow{{\bf{0}}}={\boldsymbol{\Sigma }}\overrightarrow{{\bf{c}}}\cdot (\overrightarrow{{\bf{d}}}-{\boldsymbol{\Sigma }}\overrightarrow{{\bf{c}}}),$$

where $\cdot$ denotes Hadamard (or Schur) product. Hence, we name ${\boldsymbol{\Sigma }}$ *orthogonalization operator*. If we consider ${\boldsymbol{\Sigma }}\overrightarrow{{\bf{c}}}$ as signal $\overrightarrow{{\bf{s}}}$, and accordingly $\overrightarrow{{\bf{d}}}-{\boldsymbol{\Sigma }}\overrightarrow{{\bf{c}}}$ as background noise $\overrightarrow{{\bf{n}}}$, equations (15) and (16) become the classical models used in^42–45^,

17 $$\overrightarrow{{\rm{d}}}=\overrightarrow{{\bf{s}}}+\overrightarrow{{\bf{n}}},$$

18 $$\overrightarrow{{\bf{0}}}=\overrightarrow{{\bf{s}}}\cdot \overrightarrow{{\bf{n}}}\mathrm{.}$$

Therefore, if we assume the weak signal is orthogonal to the background noise in microseismic monitoring, ${\boldsymbol{\Sigma }}\overrightarrow{{\bf{c}}}$ is an estimation of the weak signal.

## Solution of orthogonalization operator

The inversion problem in equation (13), however, is ill-posed. To stabilize the optimization, an extra regularization term is necessary to solve equation (13):

19 $${\rm{\arg }}\mathop{{\rm{\min }}}\limits_{{{\boldsymbol{\Sigma }}}_{k}}\sum _{{{\bf{c}}}_{k}\in {\bf{E}}}[{\Vert {{\boldsymbol{\Sigma }}}_{k}{{\bf{c}}}_{k}-{\bf{d}}\Vert }_{F}^{2}+ {\mathcal R} ({{\boldsymbol{\Sigma }}}_{k})],$$

where ${\mathcal R}$ represents the regularization operator. For convenience, we rewrite equation (19) as:

20 $${\rm{\arg }}\mathop{{\rm{\min }}}\limits_{{{\boldsymbol{\sigma }}}_{k}}\sum _{{{\bf{c}}}_{k}\in {\bf{E}}}[{\Vert {{\bf{C}}}_{k}{{\boldsymbol{\sigma }}}_{k}-{\bf{d}}\Vert }_{F}^{2}+ {\mathcal R} ({{\boldsymbol{\sigma }}}_{k})],$$

where ${{\bf{C}}}_{k}$ is a diagonal matrix composed by ${c}_{k}(t)$: ${{\bf{C}}}_{k}=diag({c}_{k}(t))$. ${{\boldsymbol{\sigma }}}_{k}$ is a column vector composed by ${{\boldsymbol{\sigma }}}_{k}(t)$: ${{\boldsymbol{\sigma }}}_{k}=[{{\boldsymbol{\sigma }}}_{k}(t{)]}^{T}$. Note that ${{\boldsymbol{\Sigma }}}_{k}{c}_{k}={{\bf{C}}}_{k}{{\boldsymbol{\sigma }}}_{k}={{\boldsymbol{\sigma }}}_{k}\cdot {{\bf{c}}}_{k}$.

One of the most commonly used regularization approaches is Tikhonov’s regularization^40^, in which one additionally attempts to minimize the norm of ${\bf{T}}{{\boldsymbol{\sigma }}}_{k}$, where ${\bf{T}}$ is the regularization operator^39^. The regularized problem can be expressed as:

21 $${\rm{\arg }}\mathop{{\rm{\min }}}\limits_{{{\boldsymbol{\sigma }}}_{k}}\sum _{{{\bf{C}}}_{k}\in {\bf{E}}}[{\Vert {{\bf{C}}}_{k}{{\boldsymbol{\sigma }}}_{k}-{\bf{d}}\Vert }_{F}^{2}+{\varepsilon }^{2}{\Vert {\bf{T}}{{\boldsymbol{\sigma }}}_{k}\Vert }_{F}^{2}],$$

where $\varepsilon$ is a scalar scaling parameter. The formal solution has the well-known form,

22 $${\bar{{\boldsymbol{\sigma }}}}_{k}={({{\bf{C}}}_{k}^{T}{{\bf{C}}}_{k}+{\varepsilon }^{2}{{\bf{T}}}^{T}{\bf{T}})}^{-1}{{\bf{C}}}_{k}^{T}{\bf{d}}\mathrm{.}$$

where ${\bar{{\boldsymbol{\sigma }}}}_{k}$ present a least-squares estimate of ${{\boldsymbol{\sigma }}}_{k}$. Tikhonov’s regularization can be interpreted as a roughening approach. Although Tikhonov’s regularization is effective, the parameter $\varepsilon$ and regularization operator ${\bf{T}}$ are typically difficult to choose, from the user perspectives^46,39^ proposed a particularly convenient shaping regularization approach, in which, a triangle shaping operator ${\boldsymbol{\Gamma }}$ is proposed and introduced into the iterative inversion as a fundamental operation. The relation between regularization operator **T** and shaping operator ${\boldsymbol{\Gamma }}$ can be expressed as^39^:

23 $${\boldsymbol{\Gamma }}=({\bf{I}}+{\varepsilon }^{2}{{\bf{T}}}^{T}{\bf{T}}{)}^{-1}$$

Combining equation (22) and (23), we have:

24 $${\bar{{\boldsymbol{\sigma }}}}_{k}={[{\bf{I}}+{\boldsymbol{\Gamma }}({{\bf{C}}}_{k}^{T}{{\bf{C}}}_{k}-{\bf{I}})]}^{-1}{\boldsymbol{\Gamma }}{{\bf{C}}}_{k}^{T}{\bf{d}}\mathrm{.}$$

By introducing scaling of A by $\mathrm{1/}\lambda$ in equation (24), we can rewrite it as:

25 $${\bar{{\boldsymbol{\sigma }}}}_{k}={[{\lambda }^{2}{\bf{I}}+{\boldsymbol{\Gamma }}({{\bf{C}}}_{k}^{T}{{\bf{C}}}_{k}-{\lambda }^{2}{\bf{I}})]}^{-1}{\boldsymbol{\Gamma }}{{\bf{C}}}_{k}^{T}{\bf{d}}\mathrm{.}$$

where $\lambda$ is an introduced parameter controlling the physical dimensionality and enabling fast convergence when inversion is implemented iteratively^27^.

## Implementation of the orthogonalized morphological reconstruction

The SE plays an important role in the morphological decomposition and reconstruction. The SE has three parameters: shape, height (the amplitude of SE), and width (the width of definitional domain of SE). Generally speaking, the shape of SE can be a semicircle, a triangle, or a straightline. The SEs with different parameters has different scales. When the shape of a SE is fixed, its scale increases as the height decreases (or as the width increases). A SE with a large (or small) scale indicates that it has a fat (or slim) structure (i.e., its shape is close to the shape of a constant (or $\delta$) function). The comparison of scale among the three shapes is as follow:

26 $$Scale(straightline) > Scale(semicircle) > Scale(triangle\mathrm{).}$$

In the morphological decomposition, we need a series of SE with different scales to obtain the different morphological information of the input data. For a specific morphological decomposition, a commonly used strategy to produce the SE family ${{\bf{b}}}_{K}$ is that we fix the shape of the SE and gradually increase both its height and width to produce different SEs. The rate of increase determines the performance of decomposition. Another more convenient strategy is that the $i$ th SE ${b}_{i}$ can be produced by $i-1$ times self morphological dilation:

27 $${{\bf{b}}}_{i}=\mathop{\underbrace{{\varphi }_{{b}_{1}}\mathrm{(...}{\varphi }_{{b}_{1}}({\varphi }_{{b}_{1}}}}\limits_{i-1\,times}({{\bf{b}}}_{1}\mathrm{))).}$$

An iterative optimization can greatly improve efficiency in solving an inverse problem when the computational scale is large. We choose the classical conjugate gradient method^47^ to iteratively implement the orthogonalized morphological reconstruction approach. The conjugate gradient algorithm requires symmetric positive definite operators. So the shaping operator splits into two matrices, ${\boldsymbol{\Gamma }}={\bf{H}}{{\bf{H}}}^{T}$. Equation (25) can then be written as:

28 $${\bar{{\boldsymbol{\sigma }}}}_{k}={\bf{H}}{[{\lambda }^{2}{\bf{I}}+{{\bf{H}}}^{T}({{\bf{C}}}_{k}^{T}{{\bf{C}}}_{k}-{\lambda }^{2}{\bf{I}}){\bf{H}}]}^{-1}{{\bf{H}}}^{T}{{\bf{C}}}_{k}^{T}{\bf{d}}\mathrm{.}$$

The estimated weak signal ${\bf{s}}$ by the orthogonalized morphological reconstruction can be represented as:

29 $${\bf{s}}\approx \sum _{{{\bf{c}}}_{k}\in {\bf{E}}}[{{\bf{C}}}_{k}{\bar{{\boldsymbol{\sigma }}}}_{k}\mathrm{].}$$

## Efficiency and effectiveness analysis of the orthogonalized morphological reconstruction

**Figure 3:**
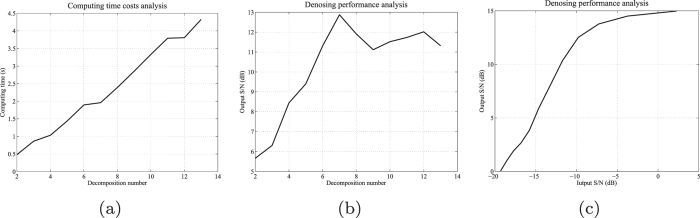
Efficiency and effectiveness analysis of the orthogonalized morphological reconstruction.

The proposed technique first decomposes the input data into a series of components with different morphological features, and then reconstructs the signal by several selected components with an orthogonalization operator. Decomposition with a higher order can obtain a more careful multi-scale morphology analysis of the input data, and accordingly is easier to separate signal and noise. Unfortunately, a large number of decompositions will pose a very expensive computational cost. Our experience shows that 4–10 decomposed components are appropriate for most seismic data sets, taking the compromise between efficiency and effectiveness into consideration. Figure 3 demonstrates an experimental analysis of the proposed orthogonalized morphological reconstruction method. Figure 3(a,b) show the computing time costs and denoising performance analysis varying with different numbers of decomposition of the input data. We can observe that, as the decomposition number increases, the computational time increases. The denoising performance of the proposed technique is reinforced as the decomposition number increases within a relatively small value (2–6), but maintains relatively stable when the decomposition number is greater than 6. Figure 3(c) shows the denoising performance varying with different input S/Ns.

## Test of the orthogonalized morphological reconstruction

**Figure 4:**
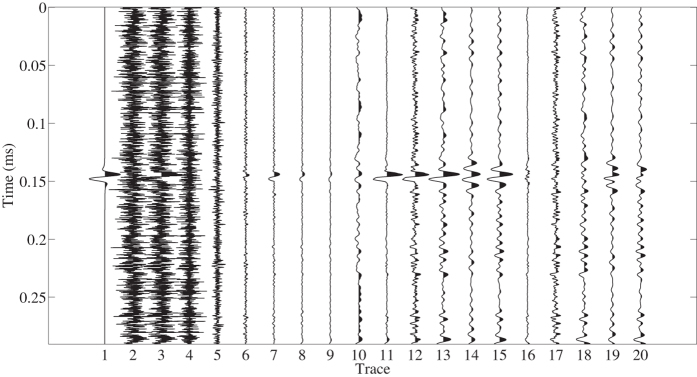
The first synthetic example. From left to right: the 1st trace: signal, the 2nd trace: Gaussian noise, the 3th trace: signal + Gaussian noise (input data), the 4th–10th traces: seven multi-scale components, the 11th trace: orthogonalized morphological reconstruction, the 12th trace: conventional morphological reconstruction, the 13th trace: result using band-pass filtering with trapezoidal band 10–20–180–190 Hz, the 14th trace: result using band-pass filtering with trapezoidal band 70–80–120–130 Hz, the 15th trace: result using band-pass filtering with trapezoidal band 60–70–170–180 Hz, the 16th–20th traces: errors of two reconstructions and three filtered results.

A synthetic signal is used to test our proposed method in this section. The first experiment is shown in Fig. 4. The synthetic signal is a Ricker wavelet with 100 Hz dominant frequency and $\pi \mathrm{/2}$ initial phase, as shown by the 1st trace. The synthetic noise is broadband Gaussian noise as shown in the 2nd trace. The 1st trace is added with the 2nd trace as the input data (the 3th trace). The S/N of the input data is $-11.6971$ dB and the definition of S/N is shown below^48^:

30 $$S/N\,=10\,\mathrm{log}\,\frac{{\Vert {\bf{s}}\Vert }_{F}^{2}}{{\Vert {\bf{n}}\Vert }_{F}^{2}},$$

where ${\bf{s}}$ is the true signal and ${\bf{n}}$ denotes the added noise. We can see that the signal is masked by the strong background noise and hardly to detect. For detection of the masked signal, the input data is decomposed into seven multi-scale morphological components as shown in the 4th–10th traces. It can be observed that most energy of noise is decomposed into the 1st and 2nd multi-scale components (the 4th and 5th traces) and the signal can be followed more or less in the rest components. Thus the 3th–7th multi-scale components (the 6th–10th traces) are used to reconstruct the signal. The results using the proposed and conventional morphological reconstruction approaches are shown in the 11th and 12th traces, respectively. The weighting coefficients in conventional approach are chosen manually as $\mathrm{(1,1,1,1,0.5)}$ associated to the 6th–10th traces taking the compromise between preserving signal and suppressing noise into consideration. It is clear that the signal is much more detectable after both two reconstructions. But the proposed approach suppress more background noise and makes the signal easier to detect than the conventional. As a comparison, the commonly used band-pass filtering is applied to the input data. The results using three different band-pass filterings (trapezoidal bands 10–20–180–190 Hz, 70–80–120–130 Hz and 60–70–170–180 Hz) are plotted in the 13th, 14th and 15th traces. The weak band-pass filtering can preserve signal well but pass a lot of background noise at the same time. The strong band-pass filtering can suppress more noise but damage the signal. The corresponding errors (differences between the true signal and processed results) associated to traces 11–15 are presented in traces 16–20 respectively. It is obvious that the proposed orthogonalized morphological reconstruction obtains the the smallest error. The S/Ns of the processed results using the proposed and conventional morphological reconstruction approaches and three band-pass filterings are $10.8905$, $-0.1852$, $-0.1788$, $0.9289$ and $0.6348$ dB, respectively. The calculation of S/N refers to equation (30), except that $n$ denotes the error. It is obvious that the proposed approach obtain the highest S/N. The cross-correlation coefficients between original signal and the five denoised signals are $0.9585$, $0.6446$, $0.7086$, $0.6163$, and $0.6913$, respectively.

**Figure 5:**
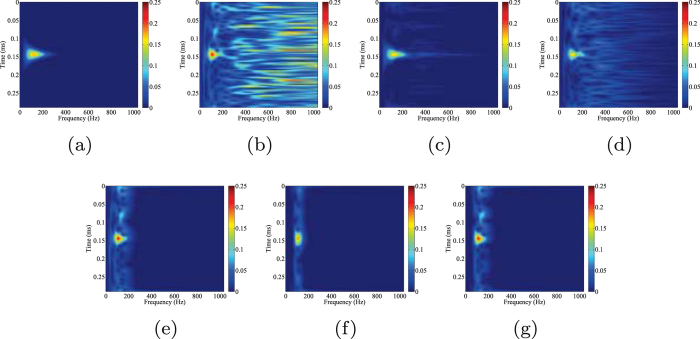
Comparison of time-frequency spectrums of (**a**) clean data, (**b**) noisy data, (**c**) orthogonalized morphological reconstruction, (**d**) conventional morphological reconstruction, (**e**) filtered data (10–20–180–190 Hz), (**f**) filtered data (70–80–120–130 Hz), (**g**) filtered data (60–70–170–180 Hz) of the first synthetic example.

Figure 5 shows time-frequency spectra of clean data, noisy data, two reconstructions and three filtered results, which give us a more detailed comparison. The time-frequency spectrum is obtained by using standard Stockwell transform^49^. As we can see from Fig. 5(a), the energy of the synthetic signal is concentrated in the area of 0.12–1.07 ms and 0–300 Hz. Contaminated by Gaussian noise (Fig. 5(b)), the time-frequency spectrum become noisy. The result using the proposed technique is shown in Fig. 5(c). An excellent reconstruction of the initial synthetic signal is obtained. Most of the noise has been attenuated and the signal’s energy is much more distinct. Figure 5(d) presents the reconstructed data obtained after using the conventional morphological reconstruction technique. Even though the quality of data is improved, the detected signal is still strongly affected by the noise. Figure 5(e,f), show the filtered data by different band-pass filters. The band-pass filtering is achieved by the low-cutoff and high-cutoff in frequency domain. As we can observe from Fig. 5(e,f), the low and high frequency components are removed but the mixed parts in the same frequency band still exist. The starting time of the detected signals in Fig. 5(d,e,f), are not as clear as that shown in Fig. 5(c), indicating that the time-picking would be better performed on the record processed by the proposed orthogonalized morphological reconstruction technique.

**Figure 6:**
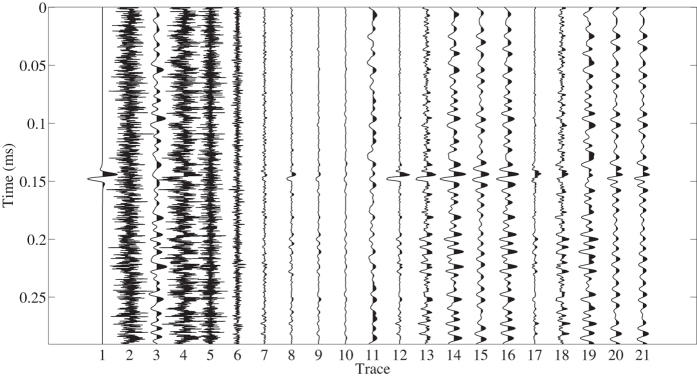
The second synthetic example. From left to right: the 1st trace: signal, the 2nd trace: Gaussian noise, the 3th trace: limited band random noise, the 4th trace: signal $+$ Gaussian noise $+$ limited band random noise (input data), the 5th–11th traces: seven multi-scale components, the 12th trace: orthogonalized morphological reconstruction, the 13th trace: conventional morphological reconstruction, the 14th trace: result using band-pass filtering with trapezoidal band 10–20–180–190 Hz, the 15th trace: result using band-pass filtering with trapezoidal band 70–80–120–130 Hz, the 16th trace: result using band-pass filtering with trapezoidal band 60–70–170–180 Hz, the 17th–21th traces: errors of two reconstructions and three filtered results.

**Figure 7:**
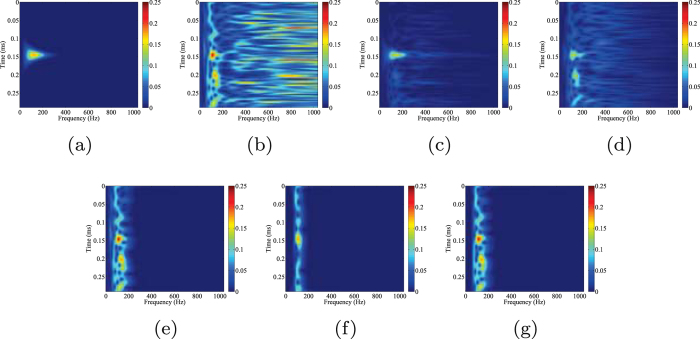
Comparison of time-frequency spectrums of (**a**) clean data, (**b**) noisy data, (**c**) orthogonalized morphological reconstruction, (**d**) conventional morphological reconstruction, (**e**) filtered data (10–20–180–190 Hz), (**f**) filtered data (70–80–120–130 Hz), (**g**) filtered data (60–70–170–180 Hz) of the second synthetic example.

The second example is demonstrated in Fig. 6. The synthetic signal (the 1st trace) is same to that in the first experiment. The added background noise consists of Gaussian noise (the 2nd trace) and limited band (40–160 Hz) random noise (the 3th trace). The input data is the sum of the 1st, 2nd and 3th traces as shown in the 4th trace. The S/N of the input data is $-12.5386$ dB. Similarly, the input data is decomposed into seven multi-scale morphological components as shown in the 5th–11th traces. In this experiment, we choose the 3 th–6th multi-scale components (the 7th–10th traces) to reconstruct the signal, taking the compromise between signal preservation and noise removal into consideration. The proposed and conventional reconstructions are plotted in the 12th and 13th traces. The weighting coefficients in conventional approach are chosen manually as $\mathrm{(1,1,1,1)}$ associated to the 7th–10th traces. It can be seen that, both approaches improve the detectability of the signal, but the proposed approach gives a better result. The three filtered data using band-pass filtering, with trapezoidal bands 10–20–180–190 Hz, 70–80–120–130 Hz and 60–70–170–180 Hz, are shown in the 14th, 15th and 16th traces. The results are unacceptable. We still hardly detect the signal in the filtered data. The S/Ns of the processed results using proposed and conventional morphological reconstruction approaches and three band-pass filterings are $4.9067$, $-2.8560$, $-6.2078$, $-3.0327$, and $-5.6471$ dB, respectively. The cross-correlation coefficients between original signal and the five denoised signals are $0.8254$, $0.4392$, $0.3944$, $0.4095$, and $0.3838$, respectively. Similarly, the time-frequency spectr a of clean data, noisy data, two reconstructions and two filtered results are shown in Fig. 7. The manually added limited-band random noise increases the difficulty of detecting the weak signal. As we can observe from Fig. 7(b), the time-frequency spectrum is extremely noisy and particularly several energy clusters in the area of 0.17–0.3 ms and 0–200 Hz can seriously obstruct the detection of the true signal. Denoising by using the orthogonalized morphological reconstruction technique leads to the results depicted in Fig. 7(c). The noise is clearly suppressed. By comparing the true signal and reconstructed signal, we can see that the two signals are very similar except for a slight amplitude damage. However, the signal would be easier to pick than before, and the slight amplitude damage is not significant, considering the totally removed noise and the observable useful signal s. The conventional morphological reconstruction approach also remove some noise, but the noise energy clusters are still noticeable. Figure 7(e,f,g) demonstrate the three filtered results. As expected, by using band-pass filtering, noise that shares the same frequency band with signal cannot be separated.

## Application to a real data set

**Figure 8:**
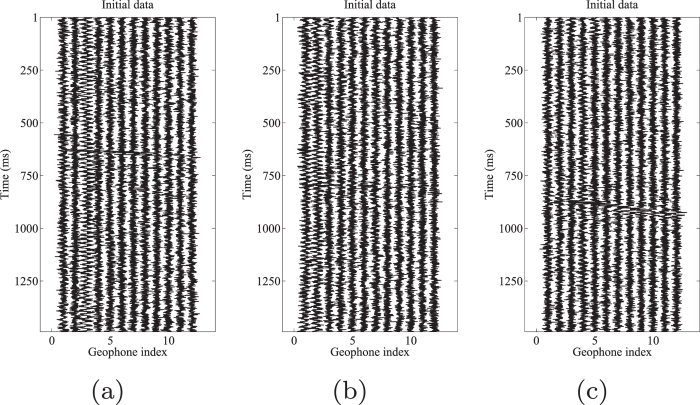
3-C microseismic data. (**a**) Horizontal components (H1). (**b**) Horizontal components (H2). (**c**) Vertical component (V).

The proposed orthogonalized morphological reconstruction is applied to a real microseismic monitoring dataset recorded in the west of China. There are twelve downhole 3-C geophones to monitor seismic activity. There are eight injection stages in this project. The magnitude of microseismic events ranges from $-3.86$ to $-0.135$ Mw. The data used in this study is produced in the last stage, in which the recording time is the longest in the whole project. Section “Supplementary material” gives the detailed information for this dataset. In this dataset, the signal induced from hydraulic fracturing is very weak when the signal reach the receivers. A lot of useful signal s cannot be detected immediately, which leads to many neglected microseismic events. Thus detection of weak signal is a vital step in this stage. A typical 1.5 s record (8631–8632.5 s after the beginning of fracturing) with horizontal components H1 and H2, vertical component V is shown in Fig. 8. As we can see from the initial data, the microseismic record is very noisy and the background noise masks the useful signals. The relative strong S wave is visible in the V component record. However, the events are difficult to follow in both H1 and H2 components. The frequency band of the perforation signal ranges from 0 Hz to 500 Hz. The frequency band of the observed microseismic signals and background noise ranges from 0 Hz to 350 Hz and from 0 Hz to 900 Hz, respectively. Due to the impact of industrial electricity, there are low-frequency interferences in several traces. The S/N of the initial dataset is approximately $-14.5942$ dB.

**Figure 9:**
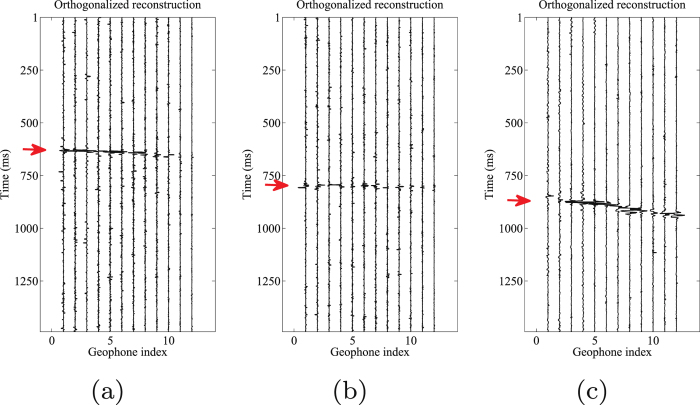
Orthogonalized morphological reconstruction results of (**a**) horizontal components (H1) by 2nd–4th scales components, (**b**) horizontal components (H2) by 2nd–4th scales components, and (**c**) vertical component (V) by 2nd–5th scales components.

We then decompose the initial data into five morphological scale components. Fig. 9 shows the results after the proposed orthogonalized morphological reconstruction approach. It can be observed that the events are much more clear than that in the raw data. We can easily follow the coherent energy in all H1 (Fig. 9(a)), H2 (Fig. 9(b)), and V (Fig. 9(c)) component records. The S/N of the denoised result is approximately $4.0927$ dB. As a comparison, the traditional morphological reconstruction approach is applied to this example. Similarly, the 2nd–4th scale components are used to reconstruct both H1 and H2 components, and the 2nd–5th scale components are used to reconstruct V components. The weighting coefficients are chosen manually as $\mathrm{(1,1,0.5)}$, $\mathrm{(1,1,0.5)}$ and $\mathrm{(1,1,1,1)}$, respectively.

**Figure 10:**
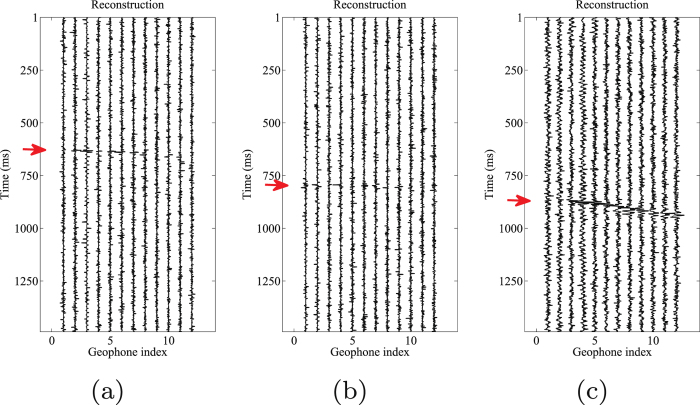
Traditional morphological reconstruction results of (**a**) horizontal components (H1) by 2nd–4th scales components, (**b**) horizontal components (H2) by 2nd–4th scales components, and (**c**) vertical component (V) by 2nd–5th scales components.

**Figure 11:**
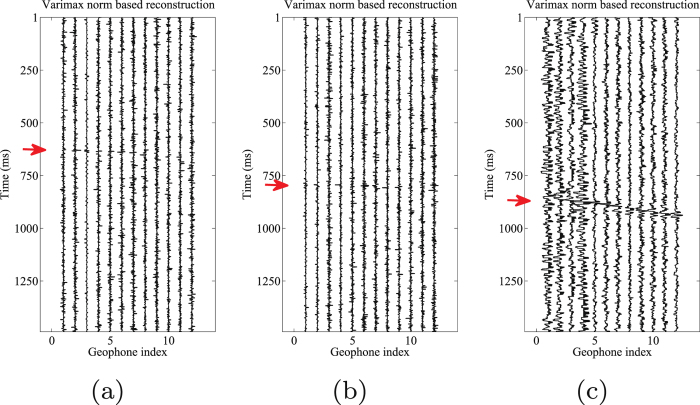
Varimax norm based morphological reconstruction results of (**a**) horizontal components (H1) by 2nd–4th scales components, (**b**) horizontal components (H2) by 2nd–4th scales components, and (**c**) vertical component (V) by 2nd–5th scales components.

The results using the traditional morphological reconstruction approach are shown in Fig. 10. The events are more visible than the initial data, but the proposed approach performs better. The S/N of the denoised result is approximately $-3.1015$ dB. In order to avoid manually choosing the weighting coefficient ${{\rm{\sigma }}}_{k}$, a varimax norm based morphological reconstruction approach can be used, in which the ${{\rm{\sigma }}}_{k}$ is defined as:

31 $${{\rm{\sigma }}}_{k}=\mathrm{1/}nor{m}_{v}({{\bf{c}}}_{k}),$$

where $nor{m}_{v}(\cdot )$ is the varimax norm^50^. The results are shown in Fig. 11. The reconstructions of the H1 (Fig. 11(a)) and V (Fig. 11(c)) components are acceptable, but the reconstruction of H2 (Fig. 11(a)) component is unsatisfied. The event is still hardly detected in the H2 component. The S/N of the denoised result is approximately $-5.1291$ dB.

**Figure 12:**
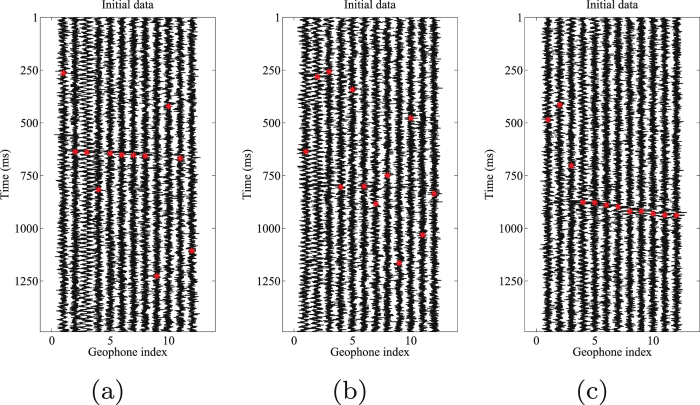
Arrival picking of the initial data. (**a**) Horizontal component (H1). (**b**) horizontal component (H2). (**c**) Vertical component (V).

**Figure 13:**
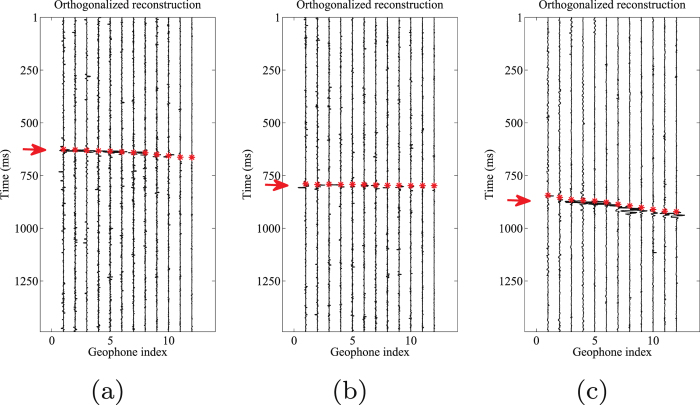
Arrival picking of orthogonalized morphological reconstructions. (**a**) Horizontal component (H1). (**b**) horizontal component (H2). (**c**) Vertical component (V).

**Figure 14:**
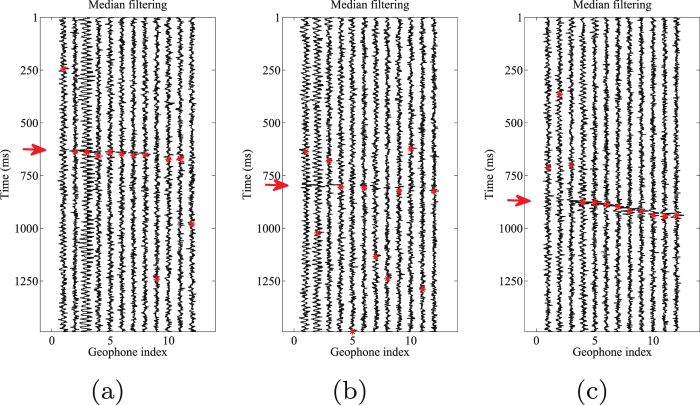
Arrival picking results using median filtering. (**a**) Horizontal component (H1). (**b**) horizontal component (H2). (**c**) Vertical component (V).

**Figure 15:**
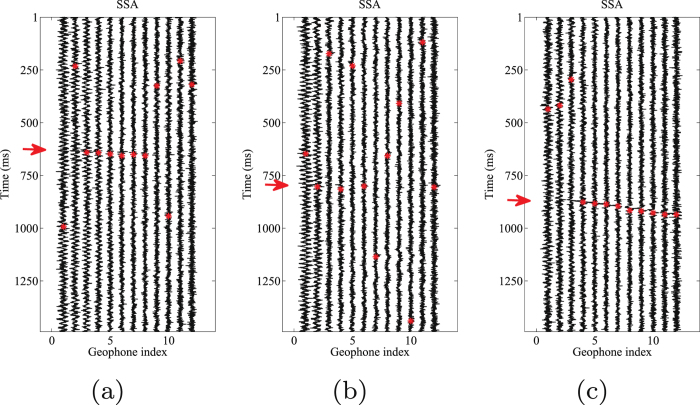
Arrival picking results using SSA. (**a**) Horizontal component (H1). (**b**) horizontal component (H2). (**c**) Vertical component (V).

Event location is an important step in the processing of microseismic data. For further evaluation of denoising performance by our proposed and other competitive approaches, we pick the events in each processed results. An accurate time-picking corresponds to a good weak signal detecting performance. The automatic events detection algorithm is chosen as the well-known STA/LTA filter^51^. In this test, the energy is used as characteristic function (CF) in STA/LTA filter. In the following figures the time picks are represented with red asterisks. Figure 12 shows the picked arrival times for the noisy 3-C records. Figures 13–15 show the results using the proposed method, median filtering and singular spectrum analysis (SSA) method, respectively. As we can see from this test, because of the strong background noise, the microseismic events are hard to pick. We can observe from Fig. 12 that STA/LTA filter is triggered at incorrect time for many traces. It is obvious that after using the proposed orthogonalized morphological reconstruction approach, the events become much more clear and easier to pick than others, which indicates the superior performance of our proposed approach.

**Figure 16:**
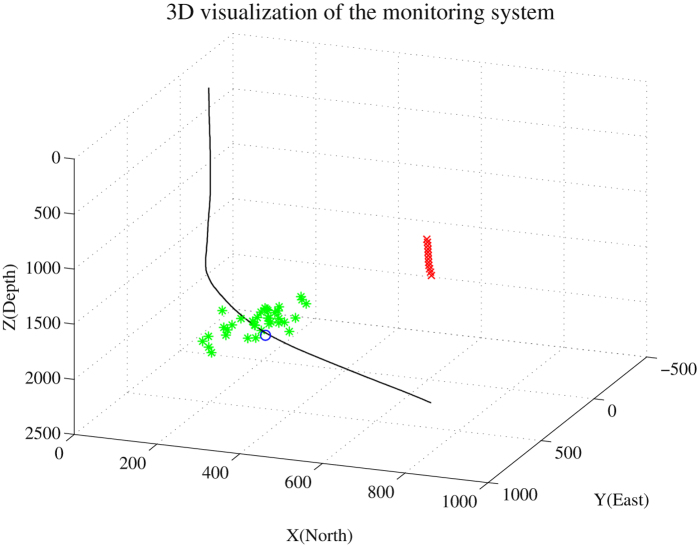
The results of source locating. The black curve line, blue circle and red crosses denote the trajectory of the fracturing well, the position of perforation and geophones, respectively. The green asterisks denote the inverted locations of the microseismic events.

We apply the STA/LTA algorithm to a longer duration of recorded data and denoised data. The experimental results as presented in Table 1 show that more events have been detected after using the proposed denoising approach than using other methods. We use the detected events in the denoised data by the proposed approach to locate the sources. We use Geiger’s approach^52^ to obtain the location. One can find the details of this approach in^53^. The calculation of travel-time is based on the principle of ray tracing. The results of locating is shown in Fig. 16. The black curve line denotes the trajectory of the fracturing well. The blue circle denotes the position of perforation. The green asterisks denote the locations of the microseismic events.

**Table 1:** Comparison of events detection in recorded data and denoised data after using different denoising approaches.

Recorded	Proposed	Median filtering	SSA
15	37	23	18

## Conclusion

We have proposed a novel denoising method based on mathematical morphological decomposition. We introduce an orthogonalization operator into the process of reconstruction, which can impel the reconstruction of weak signal. We give detailed mathematical introduction of the new method and connect it with several well-known methods and mathematical models. The most striking difference between the proposed and traditional methods is that the core calculations in the proposed method are based on logical operation and set theory. Synthetic and real data examples demonstrate its superior performance compared with the competing alternative approaches. The detected weak signals make the microseismic monitoring feasible in severe environment where the recorded data is extremely noisy and microseismic signals are very weak. The proposed orthogonalized morphological reconstruction method belongs to a class of single-channel techniques and does not require array data. It can be used not only in microseismic monitoring, but also in other type of seismic data (active source or earthquake data), and in other real world applications, e.g., image processing and signal processing, large-scale earthquake data processing and inversion. The proposed method is promising for a wide research community and industrial applications.

## Electronic supplementary material

Supplementary Material
