# Supplementary material for: Unveiling the signals from extremely noisy microseismic data for high-resolution hydraulic fracturing monitoring
Source: Sci Rep. 2017 Sep 20;7:11996. doi: 10.1038/s41598-017-09711-2 (PMC5607239; doi:10.1038/s41598-017-09711-2)
Supplement: Supplementary file 1 — Supplementary Material [file 41598_2017_9711_MOESM1_ESM.pdf]

”Unveiling the signals from extremely noisy microseismic data for high-resolution hydraulic fracturing monitoring”

by Weilin Huang, Runqiu Wang, Huijian Li and Yangkang Chen

Supplementary material

There are twelve downhole 3-C geophones to monitor seismic activity in the project. The sampling interval is 0.5 ms and the spatial increment between two geophones is 30 m. Rock in target layer is fractured by a pressurized liquid. The fracturing well is a horizontal well and the monitoring well is a vertical well. The shallowest geophone is at the depth of 1621 m and the deepest one is at the depth of 1951 m. The horizontal distance between fracturing and monitoring wells is approximately 620 m. The depth range of hydraulic fracture growth is between 2200 m and 3000 m. There are eight injection stages in this project. The data used in this study is produced in the last stage. The position of perforation is X(North)= 276.934 m, Y(East)=281.22 m, Z(Depth)= 2023.12 m. The positions of the geophones are shown in Table 1. Figure 1 shows the 3D visualization of the spatial position of the monitoring system. The black curve line denotes the trajectory of the fracturing well. The blue circle denotes the position of perforation. The positions of the twelve downhole 3-C geophones are represented by the red crosses. Figure 2 is the well logging data showing the velocities of P wave and S wave, Gr curve, Poisson ratio and Vp/Vs. The velocities of P wave and S wave range between $[4000, 6000]$ and $[2500, 3500]$, respectively. According to the logging data, we approximate geological structure between 2200 m and 3000 m as nine horizontal layers. The detailed parameters are shown in Table 2. In the eighth stage, the total monitoring time is approximately 3 hours. The predicted length and width of the fracture grid are 920 m and 450 m, respectively.

Geophone	X(North)	Y(East)	Z(Depth)	Distance
1	480.207	-450.266	1621.713	858.7894156
2	481.134	-452.162	1651.633	847.0828703
3	481.654	-452.933	1681.613	835.1753929
4	482.011	-453.342	1711.603	823.8208234
5	482.301	-453.708	1740.603	813.7018036
6	482.497	-454.063	1771.603	803.8395601
7	482.708	-454.360	1801.593	795.2955974
8	483.202	-454.638	1831.583	787.8577958
9	484.217	-455.047	1861.563	781.7646715
10	485.611	-455.778	1890.523	777.3635615
11	487.405	-456.988	1921.453	774.3289172
12	489.011	-458.101	1951.383	772.4754961

Table 1. Positions of geophones in meters. The fifth column is the distances between geophones and perforation.

Layers	P-wave velocity	S-wave velocity
2000-2098	5269	3036
2098-2197	4815	2744
2197-2357	4626	2620
2357-2483	4362	2666
2483-2546	4494	2630
2546-2696	4386	2609
2696-2831	4370	2562
2831-2900	4510	2568
2900-3000	4601	2552

Table 2. Velocity model estimated from well logging data.

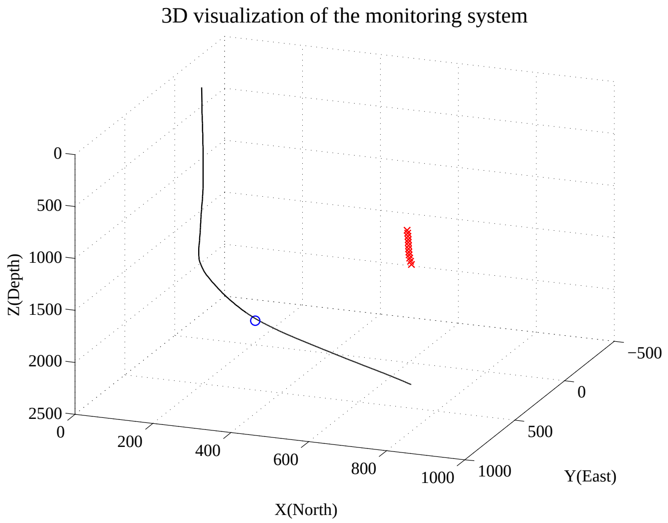

Figure 1. 3D visualization of the spatial position of the monitoring system. The black curve line, blue circle and red crosses denote the trajectory of the fracturing well, the position of perforation and geophones, respectively.

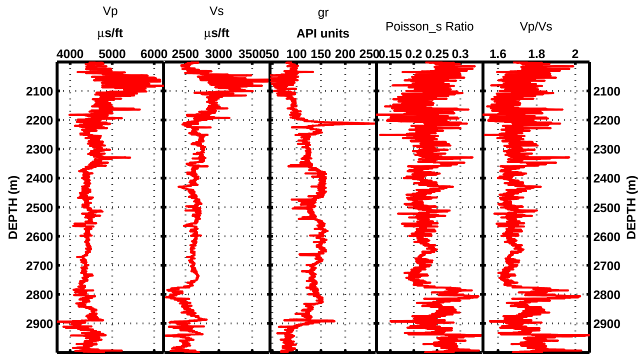

Figure 2. The velocities of P wave and S wave, Gr curve, Poisson ratio and Vp/Vs of local structure between 2000 and 3000 m.
